# TGF-β-Based Therapies for Treating Ocular Surface Disorders

**DOI:** 10.3390/cells13131105

**Published:** 2024-06-26

**Authors:** Fernando T. Ogata, Sudhir Verma, Vivien J. Coulson-Thomas, Tarsis F. Gesteira

**Affiliations:** 1 College of Optometry, University of Houston, 4901 Calhoun Road, Houston, TX 77204, USA; ftogata@central.uh.edu (F.T.O.); sverma20@central.uh.edu (S.V.); vjcoulso@central.uh.edu (V.J.C.-T.); 2 Deen Dayal Upadhyaya College, University of Delhi, Delhi 110078, India

**Keywords:** corneal wound healing, TGF-β signaling pathway

## Abstract

The cornea is continuously exposed to injuries, ranging from minor scratches to deep traumas. An effective healing mechanism is crucial for the cornea to restore its structure and function following major and minor insults. Transforming Growth Factor-Beta (TGF-β), a versatile signaling molecule that coordinates various cell responses, has a central role in corneal wound healing. Upon corneal injury, TGF-β is rapidly released into the extracellular environment, triggering cell migration and proliferation, the differentiation of keratocytes into myofibroblasts, and the initiation of the repair process. TGF-β-mediated processes are essential for wound closure; however, excessive levels of TGF-β can lead to fibrosis and scarring, causing impaired vision. Three primary isoforms of TGF-β exist—TGF-β1, TGF-β2, and TGF-β3. Although TGF-β isoforms share many structural and functional similarities, they present distinct roles in corneal regeneration, which adds an additional layer of complexity to understand the role of TGF-β in corneal wound healing. Further, aberrant TGF-β activity has been linked to various corneal pathologies, such as scarring and Peter’s Anomaly. Thus, understanding the molecular and cellular mechanisms by which TGF-β1-3 regulate corneal wound healing will enable the development of potential therapeutic interventions targeting the key molecule in this process. Herein, we summarize the multifaceted roles of TGF-β in corneal wound healing, dissecting its mechanisms of action and interactions with other molecules, and outline its role in corneal pathogenesis.

## 1. Introduction

The human cornea is a clear and avascular tissue that allows the passage of light for vision and serves as the primary refractive surface of the eye [1]. As an external tissue, the cornea offers protection to other ocular structures but is also exposed to injuries, ranging from minor scratches to deep traumas. A highly effective process of wound healing is crucial for the cornea to be able to restore its structure and function following major and minor insults. The corneal wound healing process is intricately orchestrated, with Transforming Growth Factor-Beta (TGF-β) having a central role [2]. 

TGF-β is a versatile signaling molecule that coordinates various cell responses. Upon corneal injury, TGF-β is rapidly released into the extracellular environment, triggering many cell responses that are essential for successful wound healing. TGF-β triggers corneal epithelial cell migration and proliferation, which are necessary for epithelial wound closure. TGF-β also triggers the proliferation of keratocytes and their trans-differentiation into myofibroblasts [3]. TGF-β then mediates the secretion of a provisional matrix within the wound bed by the myofibroblasts, including collagen, fibronectin, laminin, proteoglycans, hyaluronan, matrix metalloproteinases (MMPs), and tissue inhibitors of metalloproteinases (TIMPS), all vital for the wound healing process [4,5]. However, excessive levels of TGF-β can lead to abnormal collagen deposition, resulting in corneal scarring and impaired vision [2,6,7,8,9,10,11,12]. Therefore, carefully balanced TGF-β activation and regulation are crucial for the maintenance of corneal clarity and vision quality following an injury. This review seeks to provide an understanding of how TGF-β facilitates each stage of the corneal wound healing process, from initial injury response to tissue remodeling and wound resolution. By examining the roles of TGF-β in the wound healing process, including in the cellular and molecular interactions, we aim to shed light on the delicate balance required between wound resolution with the restoration of tissue function and corneal scarring. Additionally, we discuss how aberrations in TGF-β signaling can lead to various corneal pathologies and the implications this has for developing targeted therapies. Through this analysis, we hope to highlight the potential for new therapeutic approaches that could mitigate the risks of excessive scarring and improve outcomes for individuals suffering from corneal injuries.

### 1.1. TGF-β Signaling Pathway

In the context of corneal injuries, TGF-β signaling is indispensable, orchestrating the molecular and cellular pathways responsible for the restoration of corneal integrity [13]. Upon corneal injury, the rapid release of TGF-β from latent reservoirs within the extracellular matrix is triggered [2,14]. The latent form of TGF-β is cleaved into an activated state via the action of enzymes within the extracellular environment, enabling TGF-β to bind to TGF-β receptors on the cellular surface, initiating both canonical and noncanonical signaling pathways [15]. The activation of latent TGF-β upon corneal injury involves the action of specific enzymes, such as matrix metalloproteinases (MMPs) [16]. In its latent form, TGF-β is associated with the latency-associated peptide (LAP) [17,18], forming a complex that keeps TGF-β inactive and sequestered within the extracellular matrix. In response to corneal injury or other stimuli, certain MMPs, particularly MMP-9 (matrix metalloproteinase-9), are produced and activated [19,20]. These enzymes, in turn, cleave LAP, effectively releasing active TGF-β [17]. The activation of TGF-β is tightly regulated, ensuring that it is released and activated only in response to the appropriate cues, such as tissue injury or inflammation [2,14]. 

**Figure 1:**
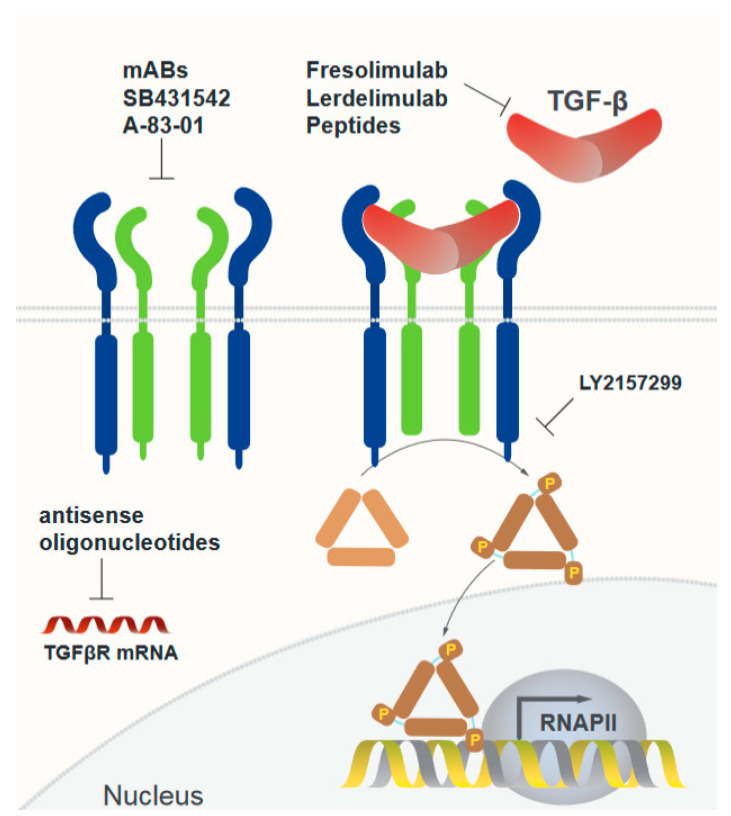
Extracellular dimeric TGF-β binds to its transmembrane receptor (TGFβR), triggering the transphosphorylation of the receptor. The activated receptor recruits and phosphorylates R-Smads, downstream proteins in the TGF-β signaling pathway. Activated R-Smads migrate to the nucleus, where they interact with other co-activators, forming the transcription complex. Three approaches have been used to disrupt this pathway as follows: (1) Monoclonal antibodies with high affinity and neutralizing capability against TGF-β. (2) Small molecules that inhibit downstream signaling from the receptor to Smads. (3) Antisense oligonucleotides developed against either TGF-β or its receptor to impair this signaling pathway.

Once activated, TGF-β engages in signaling through two main pathways—the canonical (Smad-dependent) and the noncanonical (Smad-independent) pathways. In the canonical signaling pathway, TGF-β binds to its type II receptor, which then recruits and phosphorylates the type I receptor. The activated type I receptor phosphorylates receptor-regulated Smads (R-Smads), specifically Smad2 and Smad3 [19,20]. Once phosphorylated, R-Smads form a complex with the common mediator Smad4. This complex then translocates into the nucleus where it regulates the transcription of target genes that are essential for initiating and regulating the wound healing process, including those involved in cell migration such as FN1, in cell proliferation such as CCND1 [21,22], in cell differentiation such as SNAIL1 [23,24], and in extracellular matrix production such as COL1A1, COL1A2, and COL3 [25,26]. This orchestrated gene expression response triggered by TGF-β is crucial for the proper progression of wound healing, ensuring that repair mechanisms are effectively initiated and maintained as wound healing progresses. Conversely, noncanonical TGF-β signaling pathways are Smad independent and include activating mitogen-activated protein kinases (MAPKs), such as ERK, WNT, JNK, and the p38 pathways, and/or signaling through the phosphatidylinositol-3-kinase (PI3K), and/or the AKT pathways (Figure 1). Noncanonical TGF-β signaling has been shown to contribute to the regulation of cellular processes, such as cytoskeletal reorganization, cellular motility, and the inflammatory response, all of which are also crucial for effective wound healing. TGF-β has been shown to interact with the Wnt signaling pathway, decreasing Dickkopf-1 expression and leading to enhanced Wnt pathway activity, influencing cell fate determination, particularly in the differentiation of keratocytes into myofibroblasts [27]. Activation of its type I receptor promotes the formation of a multiprotein complex capable of recruiting Ras to the cellular membrane and activating the Erk MAPK signaling pathway. This MAPK pathway influences cell proliferation, differentiation, and migration in response to TGF-β [28,29,30]. In response to TGF-β, this signaling pathway regulates epithelial to mesenchymal transition (EMT) [31,32]. This results in changes to gene transcription that are necessary for EMT, such as altering cell–matrix interactions and promoting cell motility [33,34]. Other MAPK signaling pathways activated by TGF-β are the JNK and p38 MAPK pathways, also critical for cellular responses during wound healing, contributing to the regulation of apoptosis and EMT, which are vital for tissue repair and regeneration [35,36]. Recently, a clear distinction between the canonical and non-canonical pathways was challenged, showing that the MAPK pathway can trigger SMAD2/3 activation thus leading to myofibroblast transformation [21]. Notch and TGF-β signaling have been shown to crosstalk, with a direct interaction of the Notch intracellular domain (NICD) with Smad3 [37]. This interaction influences the expression of Hes-1, a Notch target, which is essential for various cell processes, including cell differentiation and proliferation, particularly during the regeneration of the corneal epithelium [37]. Lastly, TGF-β can activate the JAK-STAT pathway, impacting the immune responses and tissue repair in the cornea. This pathway influences the production of cytokines and immune cell activation, contributing to the overall healing process. There is “crosstalk” between the different TGF-β signaling pathways, such as between SMAD, MAPK (Mitogen-Activated Protein Kinase), Wnt, Notch, and JAK-STAT (Janus Kinase-Signal Transducer and Activator of Transcription), which is critical in orchestrating cellular responses during corneal wound healing [38]. Erk activity can regulate receptor-activated Smads, and the activation of JNK and p38 by TGF-β involves interactions with other signaling molecules such as TAK1 and TRAF6, demonstrating a complex network of interactions that modulate TGF-β mediated cellular responses to injury [39,40]. Studies have also highlighted the interaction between Substance P and TGF-β in human corneal fibroblasts, showing that Substance P promotes TGF-β-induced collagen synthesis, a necessary process in corneal fibrosis [22]. 

TGF-β and bone morphogenetic proteins (BMPs) were both discovered around the same time in the 1970s. TGF-β was initially identified for its ability to stimulate the growth of fibroblast cells [23]. Similarly, the BMPs were discovered due to their ability to induce bone formation [24,25]. Since TGF-βs and BMPs signal through a similar pathway involving heteromeric membrane receptors and SMAD proteins [26], they were subsequently classified together as the TGF-β superfamily. Although the BMP and TGF-β signaling pathways have distinct ligands and receptors, studies have suggested there can be some overlap [28]. Both the BMP and TGF-β signaling pathways share some common downstream signaling components, such as SMAD 4 and 7. Specifically, BMP ligands bind and signal through BMP receptors I and II (BMPR-I and BMPR-II) and activate SMAD1/5/8, which then form complexes with SMAD4 and translocate to the nucleus to regulate gene expression. Similar to TGF-β, BMPs can also trigger non-canonical signaling pathways, such as MAPK and P13K/AKT [29]. The BMP signaling pathway is essential in maintaining tissue homeostasis and repair, and its dysregulation is implicated in numerous pathological conditions. In the context of fibrosis, BMP signaling has been shown to counteract the effects of TGF-β and inhibit fibrosis [30].

Connective Tissue Growth Factor (CTGF) is a critical downstream effector of TGF-β signaling, particularly when related to tissue fibrosis, playing a significant role in regulating extracellular matrix production, cell proliferation, differentiation, and migration [31]. The involvement of CTGF in mediating TGF-β’s effects on the extracellular matrix makes it an attractive target for therapeutic intervention in ocular surface disorders. Modulating CTGF activity could control the fibrotic response, reducing scarring and preserving vision. Peptides that mimic CTGF’s receptor-binding domain can act as competitive inhibitors, preventing CTGF from exerting its pro-fibrotic effects.

The TGF-β signaling pathway is highly controlled at various levels, including via regulation of ligand availability, via regulation of receptor activation, and via the regulation of the downstream signaling pathways such as Smad phosphorylation and translocation [32,33]. This ensures the precise modulation of the cellular responses necessary for proper wound healing and tissue repair. The pathway’s regulation involves a variety of mechanisms, including the presence of ligand antagonists, receptor inhibitors, and negative feedback loops involving inhibitory Smads (I-SMADs), which prevent excessive responses that could lead to fibrosis or other pathological conditions [27,34,35]. A subset of the Smad proteins, particularly Smad6 and 7, are known inhibitors of the TGF-β signaling pathway [36,37]. In the context of corneal injury, Smad7 activation has demonstrated promising effects in reducing the detrimental consequences of TGF-β activation. Studies have shown that animals with adenovirus-mediated overexpression of Smad7 in their corneas exhibited reduced macrophage/monocyte infiltration after alkali burns, leading to a less severe inflammatory response and decreased myofibroblast presence [38]. Further research by Myrna and collaborators [39] suggests that Smad7 expression is enhanced on patterned surfaces compared to planar ones following TGF-β exposure. Their results correlate the rich topological environment of the healthy cornea to myofibroblast transformation prevention. Understanding these mechanisms of regulation is paramount for developing therapeutic interventions to promote proper wound repair and prevent fibrosis [40] in the cornea. Disruptions in this crosstalk can lead to tissue remodeling abnormalities and scarring [41]. 

### 1.2. Receptors That Mediate TGF-β Signaling

The interaction between TGF-β ligands and their receptors determines the initiation of either canonical or non-canonical signaling pathways, with a diverse array of receptors and co-receptors that can mediate the signaling cues by TGF-β ligands thus underscoring the complexity of TGF-β signaling [42,43,44]. The primary cell surface receptors for TGF-β ligands are the TGF-β Type I Receptor (TGF-βRI or ALK5) and TGF-β Type II Receptor (TGF-βRII) [45,46,47,48]. TGF-βRI is not directly involved in the primary signal transduction and plays a collaborative role, working in tandem with TGF-βRII. The TGF-β Type I (TGF-βRI) and Type II (TGF-βRII) receptors are central for TGF-β signaling, functioning through the formation of a receptor complex. TGF-βRII is the primary binding site for TGF-β ligands [49,50]. Upon ligand binding, TGF-βRII recruits and phosphorylates TGF-βRI, also known as ALK5 [49], initiating signal transduction [51,52]. Once TGF-βRI is activated by TGF-βRII, it phosphorylates Smad proteins that modulate gene expression [53]. The TGF-β superfamily includes three primary TGF-β isoforms as follows: TGF-β1, TGF-β2, and TGF-β3 [54], each presenting differing affinity to each receptor [55,56,57]. TGF-β signaling via the TGF-βRI–TGF-βRII receptor complex is central to numerous biological processes; thus, over the years, there has been a keen interest in developing therapeutic interventions to disrupt the formation this receptor complex for treating cancer [58,59] and certain autoimmune diseases [60,61]. In the cornea, there is also great interest in disrupting the formation of this receptor complex for preventing excessive TGF-β signaling that can lead to corneal scarring [62,63]. 

Other ALKs, such as ALK1, ALK2, ALK3 (BMPR-IA), and ALK6 (BMPR-IB) receptors, are involved in TGF-β signaling, displaying roles both distinct from and overlapping with those of ALK5. ALK1, particularly noted in endothelial cells, engages in TGF-β signaling in a manner that can either complement or antagonize ALK5 signaling. It is especially involved during angiogenesis processes such as vascular development and homeostasis, where its signaling can be modulated by ligands such as BMP9. ALK2’s role in TGF-β signaling, particularly through BMP9-induced phosphorylation of Smad1/5, suggests its involvement in the proliferation and migration of cells, including cancer cells. Its aberrant expression is linked to various diseases, underscoring its potential as a therapeutic target. ALK3 and ALK6 are also engaged in BMP signaling and share properties such as BMP-dependent activation of Smad1/5/8 with ALK1 and ALK2. To date, the detailed involvement of ALK 1–3 and 6 receptors in corneal TGF-β signaling during homeostasis and in pathology remains to be studied.

Betaglycan (TGF-βRIII) can act as a co-receptor for TGF-β, presenting TGF-β to TGF-βRII, facilitating the signal transduction that is crucial for various cellular processes. Although betaglycan does not have a cytoplasmic signaling domain, it plays a significant role in regulating TGF-β signaling by presenting the TGF-β ligands to TGF-βRII, which is essential for the subsequent recruitment and activation of TGF-βRI. This interaction is critical for the phosphorylation and activation of receptor-regulated SMAD proteins and subsequent signal transduction [64,65]. Endoglin, also known as CD105, can act as a co-receptor of TGF-β in collaboration with TGF-βRII, regulating the downstream effects primarily of TGF-β1 and TGF-β3. TGF-β ligands bind and signal through endoglin via the ALK5–Smad2/3 pathway, essential branches of the TGF-β signaling cascade. This interaction regulates cellular functions such as adhesion, migration, proliferation, and apoptosis, particularly in endothelial cells which are abundant in proliferating and healing tissues [66,67]. The potential interaction between Endoglin and the Epidermal Growth Factor Receptor (EGFR) introduces an interesting aspect of crosstalk between the serine/threonine kinase signaling of TGF-β and the tyrosine kinase signaling of EGFR. It has been recently suggested that TGF-β can interact with other signaling pathways, including those mediated by EGFR, to modulate a variety of cellular responses [68].This kind of signaling crosstalk is a promising area for targeted therapeutic interventions, especially in cancer treatment where the regulation of cell growth and survival is fundamental [29,68].

Integrins can also interact with TGF-β. The collaboration between integrins and TGF-β is particularly influential in signaling via noncanonical pathways. These pathways, distinct from the traditional Smad-mediated TGF-β signaling, are instrumental in governing cellular behaviors like adhesion and migration, actions that are paramount during tissue repair and regeneration. However, when dysregulated, these pathways can drive pathological processes, leading to excessive scarring and fibrosis in the cornea. Several small molecule inhibitors targeting integrin subunits have been explored for their potential to attenuate integrin–TGF-β interactions. By binding to specific domains on integrins, these inhibitors can modulate their activity, preventing them from effectively engaging with TGF-β. This disruption can attenuate downstream noncanonical signaling, potentially curbing the fibrotic response in the cornea. Monoclonal antibodies against specific integrin subunits represent another promising strategy. These antibodies, tailored to recognize and bind to integrins, can neutralize their function. In doing so, they can disrupt the integrin–TGF-β interaction at its inception, offering a targeted approach to manage corneal fibrosis [69]. Peptides mimicking integrin-binding sequences have also shown some potential. These peptides can competitively inhibit the binding sites on TGF-β or integrins, essentially acting as decoys and preventing the two molecules from interacting.

### 1.3. TGF-β Signaling in the Cornea and Its Role in Inflammation and Corneal Pathogenesis

In the healthy cornea, TGF-β isoforms, primarily TGF-β1 and TGF-β2, along with their receptors, TGF-βRI, TGF-βRII, and TGF-βRIII, are constitutively expressed at low levels, contributing to the maintenance of corneal clarity and structural integrity. The expression of TGF-β isoforms differs in different layers of the cornea. For example, in the uninjured healthy cornea of mice, the intracellular TGF-β1 isoform is detected in the corneal epithelium, but extracellular secreted TGF-β1 is not observed; however, both TGF-β2 and TGF-β3 are detected in the extracellular environment [13]. Following an injury, the expression of TGF-β isoforms changes significantly with TGF-β1 observed as the minor isoform, while TGF-β2 is detected in large amounts and TGF-β3 is not detected at all in the corneal epithelium and stroma. However, the immunohistochemical studies on chick cornea by Huh et al. suggests that after an injury, TGF-β1 is strongly expressed in the Bowman’s layer (BL) [70]. TGF-β3 is confined to basal cells in the uninjured and regenerating regions but not detected in migrating epithelial, stromal, or endothelial cells, whereas TGF-β2 is strongly expressed in migrating and proliferating epithelial cells, active migrating fibroblasts at the wound edge, endothelial cells, and Descemet’s membrane (DM) [70]. In rabbits, TGF-β1 and TGF-β2 levels are found to be significantly higher in the anterior stroma in the initial days after a photorefractive keratectomy (PRK), compared to the naïve controls, suggesting the vital role of TGF-β in initiating the corneal wound healing response [71,72]. Thus, species-specific variation also seems to exist in TGF-β expression both in the healthy and injured corneas, which can be attributed to structural/compositional differences in the corneas [72,73]. The spatio-temporal difference in the occurrence of TGF-β isoforms in the pre- and post-injury corneal layers underscores their different roles. In healthy, uninjured corneas, low levels of TGF-β receptors are expressed across the cornea, with higher levels in the limbal epithelium. Following an injury, TGF-βRI and TGF-βRII are upregulated with increased TGF-βRII in the epithelial cells that migrated to cover the wounded area, whereas increased TGF-βRI is found across the entire corneal epithelium [74].

TGF-β also has a well-established role in modulating immune responses. In the cornea, TGF-β suppresses excessive inflammation post-injury. TGF-β has been shown to suppress the production of pro-inflammatory cytokines, such as interleukin-1 (IL-1) and tumor necrosis factor-alpha (TNF-α). By suppressing the production of these pro-inflammatory signals, TGF-β helps maintain a controlled and less inflammatory environment in the cornea [75]. TGF-β also plays a role in the recruitment and activation of regulatory T cells (Tregs) in the cornea [76]. Tregs are a specialized subset of immune cells that have immunosuppressive functions, helping to prevent excessive immune responses and promote immune tolerance [77]. TGF-β’s ability to facilitate the recruitment and activation of Tregs contributes to the regulation of inflammation in the cornea post-injury [13,78]. TGF-β can also stimulate the production of anti-inflammatory mediators, such as interleukin-10 (IL-10) [79]. IL-10 is known for its anti-inflammatory properties and can help counterbalance pro-inflammatory signals. Additionally, TGF-β can inhibit the activation and function of neutrophils, which are involved in the initial stages of inflammation [80]. This inhibition helps prevent an excessive neutrophil response, which can lead to tissue damage. Lastly, TGF-β has been found to promote the production of anti-inflammatory proteins called lipoxins, which play a role in resolving inflammation and promoting tissue repair [81,82].

TGF-β signaling must be tightly regulated since its dysregulation can lead to pathological outcomes, such as corneal fibrosis. This response is usually characterized by excessive collagen deposition and matrix metalloproteinases (MMPs) dysregulation, leading to a loss of corneal transparency and visual impairment. An imbalance and/or dysregulation of TGF-β signaling pathways have also been linked to anterior segment dysgenesis [83,84,85]. Research in humans and animals has shown that the dysregulation of TGF-β signaling leads to Peter’s Anomaly [84]. Peter’s Anomaly is a rare condition caused by the abnormal development of the cornea, leading to various corneal defects including corneal opacity and corneal and iris adhesion [86]. In fact, various mutations that lead to a loss of function of major components of the TGF-β signaling pathway result in corneal dysgenesis, including Peter’s Anomaly [83,84,85,87]. Furthermore, TGF-β signaling alterations have been linked to various other corneal pathologies, such as keratoconus, characterized by corneal thinning and steepening, and corneal ulceration, which can arise from excessive TGF-β activation in response to microbial infections [88,89]. The multifaceted role of TGF-β within the corneal microenvironment presents a unique opportunity for therapeutic intervention in a spectrum of corneal disorders. By elucidating specific signaling pathways involved in each pathological process, we can develop a strategic use of TGF-β inhibitors that can suppress inflammation and potentially promote corneal regeneration [44,72,75]. Although preclinical evidence is encouraging, further research and clinical trials are necessary to fully validate the safety and efficacy of these novel therapeutic approaches. 

TGF-β is also a key factor in corneal disorders such as Stevens–Johnson syndrome (SJS), chemical burns, and limbal stem cell deficiency. Stevens–Johnson syndrome/toxic epidermal necrolysis (SJS/TEN) is a life-threatening inflammatory mucocutaneous drug reaction characterized by erosion of the mucous membranes and skin, due to keratinocyte apoptosis [90]. SJS/TEN-associated keratocyte apoptosis also affects the ocular surface epithelium with severe outcomes [91]. The upregulated mRNA expression of TGF-β1 and TGF-βRII in the conjunctiva and elevated level of TGF-β1 cytokine in the tears of the SJS patients [92] suggest the involvement of TGF signaling in this disorder and thus a potential target for therapeutic intervention. 

Corneal chemical burns, particularly alkali burns, are a serious clinical problem, often leading to permanent visual impairment/loss. During corneal alkali injury, TGF-β1 is upregulated and promotes migration of corneal epithelial cells and keratocytes and induces trans-differentiation of the keratocytes to myofibroblasts, contributing to wound repair. But overexpression of TGF-β1 can be deleterious to the injured cornea due to haze development. Thus, regulation/inhibition of TGF-β1 expression and signaling is imperative to obtain a positive corneal wound healing and a negative corneal haze formation outcome. Chen et al. (2010) have shown that post-transcriptional regulation of TGF-β1 by GB1201, a pyrrole–imidazole (PI) polyamide prevented scarring and accelerated wound healing post alkali burn in rats [93]. TGF-β1 siRNA loaded in polyethyleneimine nanoparticles have been shown to suppress the TGF-β1 gene and ECM deposition in isolated human corneal fibroblasts. These nanoparticles can suppress the proliferation, transformation of fibroblasts to myofibroblasts and α-SMA expression [94]. 

The chemical injuries and Stevens–Johnsons syndrome are also the common causes of unilateral or bilateral limbal stem cell deficiency (LSCD), respectively. LSCD is a rare, progressive and ultimately blinding disease caused due to the depletion or dysfunction of limbal epithelial stem cells. Various surgical techniques are used for LSCD treatment, one of which is cultivated limbal epithelial transplantation (CLET). Mikhailova et al. (2014) have generated relatively pure populations of corneal epithelial-like progenitor cells from human induced pluripotent cells (hiPSCs), which are capable of terminal differentiation towards mature corneal epithelial-like cells, without the use of feeder cells or serum. To achieve this, they replicated early developmental mechanisms by blocking the TGF-β and Wnt-signaling pathways and activating fibroblast growth factor (FGF) signaling [95]. In another study, Hu et al. (2019) showed that the expansion and maintenance of primary corneal epithelial stem/progenitor cells requires the inhibition of TGFβ-RI-mediated signaling [96]. Thus, in partial LSCD corneas, inhibition of TGF-β signaling might preserve the remaining LESCs.

### 1.4. Contribution of TGF-β following Post-Refractive Surgery

Corneal haze is a common complication post-refractive surgery, such as photorefractive keratectomy (PRK), laser-assisted in situ keratomileusis (LASIK), and small incision lenticule extraction (SMILE) [97]. Unlike the clinically insignificant and corneal fibroblast-driven ‘mild haze’ that occurs in nearly all corneas following refractive surgery, the pathological ‘late haze’ that occurs in some cases has been attributed to the presence of myofibroblasts and excessive extracellular matrix (ECM) production [98,99]. Thus, many recent studies have focused on analyzing the expression levels of TGF isoforms post these refractive surgeries. In rabbits, the mRNA expression of TGF-α (in corneal epithelium) and TGF-β1 (both in corneal epithelium and stroma) increases after PRK and contributes to haze development [10]. Kaji and colleagues reported an increased expression of TGF-β1 in keratocytes four weeks after PRK that positively correlates with the appearance of corneal haze [100]. Further, increased levels of TGF-β1 have been detected in the tear fluid of patients following the excimer laser PRK [101]. Curiously, lower amounts of TGF-β1 have been reported in the tear fluid following laser subepithelial keratomileusis (LASEK) in the early postoperative days, which correlates with a lower grade of corneal haze when compared to PRK [102]. A randomized controlled trial by Long and colleagues showed decreased corneal haze after epithelial laser in situ keratomileusis (epi-LASIK) when compared to LASEK [103]. A positive correlation was found between tear TGF-β1 levels on the first postoperative day and the degree of corneal haze post one month of surgery [103].

Resolution of PRK-associated ‘late haze’ and restoration of refractive correction can be achieved by the removal of myofibroblasts by trans-differentiation and/ or apoptosis, and reabsorption of excessive ECM. Mitomycin-C is a widely used anti-haze agent, used to prevent and treat post-PRK haze in clinics [104,105], although it is associated with complications [105,106]. The topical application of anti-TGFβ blocking antibodies has shown reduced post-PRK corneal haze in rabbits [107]. Trichostatin-A, a histone deacetylase inhibitor, has also been shown to inhibit TGF-β1-induced ECM accumulation and myofibroblast generation in the human corneal fibroblasts in vitro and significantly decreased haze in the rabbit cornea in vivo [108]. Mannose-6-phosphate has also been shown to significantly reduce TGF-β1-mediated human corneal fibroblast to myofibroblast transformation in vitro, suggesting the potential to reduce haze following refractive surgery [109].

### 1.5. Therapeutic Interventions for Regulating the TGF-β Signaling Pathway

Given the central role of TGF-β in various major biological processes, a range of therapeutic approaches targeting the TGF-β ligands, their receptors, and the subsequent downstream signaling pathways have been developed with the goal of modulating TGF-β activity (see Table 1 and Figure 1). These strategies range from the use of monoclonal antibodies to small molecule inhibitors. Herein, we describe the different TGF-β based therapies that have been developed to date.

### 1.6. Monoclonal Antibodies and Fusion Proteins

Monoclonal antibodies targeting TGF-β ligands, including fresolimumab and lerdelimumab, have been investigated for use in various pathologies where TGF-β plays a critical role in disease progression [135]. These pathologies include fibrosis, cancer, and certain autoimmune diseases [136,137,138]. Fresolimumab has been studied in clinical trials for conditions such as idiopathic pulmonary fibrosis and renal fibrosis, demonstrating some potential in reducing fibrotic activity [139,140]. Lerdelimumab has been explored for its effects in scleroderma, a disease characterized by skin and organ fibrosis [141], with potential use during glaucoma surgeries [142] and reported therapeutic potential benefit to cataract patients [143].

Despite their potential, these antibodies face significant limitations. One of the main challenges is the broad role of TGF-β in immune regulation and tissue homeostasis, which can lead to adverse effects when its activity is inhibited. For instance, systemic inhibition of TGF-β might impair wound healing or enhance the risk of developing certain cancers, as TGF-β also has tumor suppressor functions in early cancer stages. Moreover, the efficacy of these treatments can vary significantly between individuals and disease subtypes, complicating the development of universally effective therapies. Further, the monoclonal antibody TRC105 (carotuximab) was developed to specifically target endoglin [144]. Although primarily tested for tumor angiogenesis, its potential application in modulating corneal cell behavior and wound healing is an avenue worth exploring. Additionally, fusion proteins such as AVID200 function similarly to the monoclonal antibodies, binding and neutralizing TGF-β ligands, leading to an inhibition of their signaling mechanisms [145,146,147].

Fusion proteins like AVID200, which act similarly to monoclonal antibodies by binding and neutralizing TGF-β ligands, have been studied in various therapeutic contexts, particularly in addressing fibrotic diseases and certain cancers. AVID200, for example, has been evaluated for its effectiveness in treating conditions such as idiopathic pulmonary fibrosis and myelofibrosis, where excessive fibrosis is a major pathological feature. The protein has shown promise in selectively inhibiting TGF-β1 and TGF-β3, which are heavily implicated in the fibrotic processes, potentially offering a more targeted approach with reduced side effects compared to broader TGF-β inhibition [148]. 

However, the application of these fusion proteins also comes with limitations. The specificity of AVID200 and similar agents might limit their usefulness in conditions where multiple TGF-β isoforms play a role. Additionally, like monoclonal antibodies targeting TGF-β, there are concerns regarding the disruption of normal TGF-β functions in tissue homeostasis and immune regulation. Such disruptions can lead to adverse effects, including compromised immune responses and abnormal tissue growth. The balance between therapeutic efficacy and potential side effects remains a significant challenge in the clinical development of these fusion proteins.

### 1.7. Peptide-Based Therapies

Peptides have been developed based on the TGF-β/receptor binding interface, such as P144 and P17 [149,150,151]. These peptides were developed as competitive inhibitors of TGF-β receptor binding, having the ability to directly disrupt the binding of TGF-β ligands to their specific receptors. Peptide inhibitors have been used to treat fibrosis [149], which is crucial in diseases like idiopathic pulmonary fibrosis, hepatic fibrosis, and scleroderma [152]. Despite their potential benefits, these peptides face several limitations. Their efficacy can be variable across different types of tissues and stages of disease due to the complex role of TGF-β in various cellular processes. Moreover, the stability and delivery of peptide-based therapies can pose significant challenges. Peptides generally have shorter half-lives in the body and may require modifications or special delivery systems to enhance their stability and ensure effective targeting to the site of disease. Additionally, there is the risk of immune responses against peptide therapeutics, which can reduce their effectiveness and safety.

### 1.8. Antisense Oligonucleotides and siRNA

Short synthetic strands of DNA, also referred to as antisense oligonucleotides, have been developed to target the mRNA of specific TGF-β receptors, with the goal of preventing their translation and subsequent expression [153,154]. For example, Trabedersen (AP 12009) targets TGF-β2 mRNA to prevent its translation, effectively reducing TGF-β2 receptor protein levels in treated cells [131,155]. Other antisense oligonucleotides have been designed against TGF-β1 and TGF-β3 receptors, showing potential in various therapeutic applications [156,157,158,159]. siRNA and antisense oligonucleotides have also been developed to downregulate endoglin expression [160]. Studies involving antisense oligonucleotides and siRNAs targeting TGF-β receptors and endoglin have demonstrated variable efficacy in different experimental and clinical settings. Trabedersen, for instance, has been studied in clinical trials for treating malignant gliomas, where it was found to reduce TGF-β2 levels and potentially improve patient outcomes by inhibiting tumor growth and reducing immunosuppression in the tumor microenvironment [131,161,162]. Other antisense oligonucleotides targeting TGF-β1 and TGF-β3 receptors have been explored in preclinical models for pulmonary and renal fibrosis, showing potential in reducing fibrotic tissue formation. Additionally, siRNAs designed to downregulate endoglin expression have been investigated primarily in the context of cancer, particularly for their role in inhibiting tumor angiogenesis, a critical process for tumor growth and metastasis. However, the clinical efficacy of these therapies has been limited by several factors. One major limitation is the delivery of these molecules to target tissues, as they need to penetrate cells and remain stable long enough to exert their effects. Additionally, off-target effects and potential toxicity pose significant concerns, as the suppression of TGF-β signaling can have wide-ranging effects on normal cellular functions, including immune regulation and wound healing. The specificity and dosing strategies of these treatments continue to be critical areas of research to enhance their therapeutic profiles and minimize adverse effects [163].

### 1.9. Small-Molecule Inhibitors

A small molecule inhibitor is a low molecular weight compound that can enter cells easily and specifically interfere with the activity of one or more proteins by binding to them. These inhibitors are often designed to block enzyme activities, receptor–ligand interactions, or protein–protein interactions critical for cellular processes. Small-molecule inhibitors, such as the widely used ALK inhibitor SB431542 and LY2109761, specifically inhibit the kinase activity of ALK5 thereby preventing the autophosphorylation of the receptor, a critical step necessary for the receptor complex formation and subsequent signaling [164,165]. SB431542 has been shown to reduce collagen expression in a rabbit model of corneal fibrosis [44]. Additionally, small molecule inhibitors targeting the interaction between endoglin and TGF-β ligands include compounds like AVID200, which is designed to selectively neutralize TGF-β1 and TGF-β3, playing a significant role in fibrosis and cancer pathologies (see above for details).

The efficacy of these small molecule inhibitors can be substantial, as they allow for targeted therapy with the potential for oral administration, which is less invasive than treatments like monoclonal antibodies. However, limitations include potential off-target effects, which can lead to unintended side effects due to the inhibitors affecting similar enzymes or receptors not intended to be targeted. Furthermore, developing resistance to these inhibitors can occur in cancer therapy, where tumor cells adapt to bypass the inhibited signaling pathways. Thus, ongoing research is crucial to improving the specificity and reducing resistance to these therapies.

Recent work by the Wilson lab has highlighted the potential of losartan, an angiotensin II receptor antagonist, as a therapeutic agent against fibrosis. Losartan has been shown to inhibit TGF-β signaling by blocking the downstream activation of ERK1/2. This inhibition leads to the apoptosis of myofibroblasts, which are key players in fibrosis. In corneal injury models, topical application of losartan has demonstrated the following two-stage process in fibrosis prevention: the first stage involves the removal of myofibroblasts through apoptosis, and second phase involves the recruitment of fibroblasts to reorganize the extracellular matrix. This dual action not only reduces scarring but also promotes proper wound healing, making losartan a promising candidate for treating fibrotic ocular disorders [166].

### 1.10. Natural Ligands

Studies have also demonstrated that various natural ligands of TGF-β ligands and TGF-β receptors have the potential to be used to modulate TGF-β activity. For example, galunisertib (LY2157299 monohydrate) inhibits TGF-β receptor activity, specifically targeting TGF-β receptor type I, also known as ALK5 [115]. Galunisertib inhibits TGF-β receptor type I kinase activity, thereby impeding the transduction of TGF-β signaling [116]. Decorin, a small leucine-rich proteoglycan, has emerged as a critical modulator of TGF-β activity, offering a novel therapeutic approach to managing conditions characterized by excessive TGF-β signaling [167]. This proteoglycan naturally binds to TGF-β, effectively sequestering the cytokine and preventing its interaction with cellular receptors. Mohan’s group elucidated the therapeutic potential of decorin, particularly in the context of ocular diseases, demonstrating that topical application of decorin can significantly reduce corneal fibrosis and enhance wound healing, thereby improving corneal transparency and overall eye health [16,72,168,169,170]. These findings underscore the feasibility of using decorin-based therapies to modulate pathological TGF-β activity effectively. While the therapeutic applications of decorin show considerable promise, there are inherent challenges such as achieving targeted delivery to the affected tissues and maintaining effective concentrations at the site of pathology. Moreover, the complex role of TGF-β in different cellular contexts may lead to variable responses among patients, necessitating further research to optimize and individualize decorin-based treatment strategies. Natural compounds, such as curcuminoids [171] and flavonoids [172], have also been found to inhibit endoglin expression, and their potential ophthalmic applications are currently under investigation [173]. Their potential therapeutic applications are being explored, although challenges such as variable patient responses and bioavailability persist, necessitating further research to optimize their clinical use [172].

## 2. Therapeutic Targets for TGF-β Signaling Based Therapies

Given their central roles in this fibrotic process, both ALK1 and ALK2 are viewed as promising therapeutic targets. Inhibiting their activity could potentially attenuate the TGF-β-driven pro-fibrotic effects, restoring a balance to the corneal tissue’s healing response. Research has suggested that inhibiting ALK1 can reduce the pro-fibrotic effects of TGF-β, including in the cornea [174]. By targeting ALK1, one can potentially suppress the excessive cellular proliferation and extracellular matrix deposition that characterizes corneal fibrosis. Further, combinatory treatment approaches could consider targeting both ALK1 and ALK2 simultaneously, creating a potential to achieve a more holistic management of corneal fibrosis, ensuring wound healing without the debilitating scars that impede clear vision.

Within the complex downstream TGF-β signaling cascade, TGF-β Activating Kinase 1 (TAK1), a mitogen-activated protein kinase (MAP3K), has been shown to have a critical role. Functioning downstream of the TGF-β receptors, TAK1 is central to noncanonical signaling pathways, influencing a variety of cellular processes beyond the traditional Smad-dependent routes. Given its essential role, particularly in the regulation of corneal cell behavior, TAK1 has emerged as a potential target for therapeutic interventions aimed at modulating the TGF-β pathway. Strategies include the development of small molecule inhibitors and peptide-based therapies to hinder TAK1 activation and its downstream signaling, thereby potentially mitigating TGF-β’s adverse effects in corneal pathology. Small molecule inhibitors targeting TAK1 are under investigation for their potential to disrupt its kinase activity. These compounds aim to selectively bind to TAK1, preventing its activation and subsequent downstream signaling. Such targeted inhibition can attenuate the pro-fibrotic and pro-inflammatory effects of TGF-β in the cornea, promoting a more controlled and physiological healing response. Peptide-based therapies have also been designed to interfere with the interaction between TAK1 and its binding partners, effectively halting the initiation of downstream signaling cascades. By doing so, they could provide a means to regulate the noncanonical pathways influenced by TAK1.

Given the significance of the interplay between TGF-β signaling and the EGFR, disrupting the crosstalk between TGF-β and EGFR has also emerged as a promising therapeutic strategy [175,176]. Several potential drugs have been investigated for their ability to modulate this interaction, aiming to mitigate the adverse effects of overactive signaling and promote controlled, physiological wound healing [177,178,179]. Tyrosine kinase inhibitors (TKIs) are a class of drugs that have been explored for their potential to target EGFR directly. By inhibiting the tyrosine kinase activity of EGFR, these drugs can reduce its activation and downstream signaling, indirectly affecting the crosstalk with TGF-β [180,181]. Erlotinib and gefitinib are examples of TKIs that have been studied for their effects on EGFR and their interaction with TGF-β signaling in various cellular contexts [182]. Their potential in modulating corneal fibrosis remains an area of active research [183,184,185]. Dual inhibitors targeting both the TGF-β and EGFR pathways simultaneously represent another exciting avenue [186,187]. These drugs aim to offer a broader spectrum of action by dampening the synergistic effects of both pathways, thereby minimizing the risk of excessive fibrosis during corneal wound repair [188]. Monoclonal antibodies against EGFR, such as cetuximab, have also been considered. These antibodies can specifically bind to EGFR, preventing its activation and, consequently, its interaction with TGF-β [189].

Zamudio and collaborators have proposed the use of TGF-β inhibitors (SB431542 or A-83-01 [190]) as a means to promote stem cell proliferation [191]. Limbal stem cell deficiency (LSCD) is a devastating corneal pathology that arises from the depletion of limbal epithelial stem cells (LESCs) that reside in the limbus and are crucial for maintaining corneal epithelial turnover and transparency. By inhibiting TGF-β, these treatments could facilitate the regeneration of LESCs, thereby promoting corneal repair and restoring transparency. 

In a study of corneal transplantation, TGF-β has been shown to play a crucial role in triggering the immune response that can lead to graft rejection by reducing the density of endothelial cells [192]. To address this, the authors utilized an in vitro treatment scheme with a TGF-β neutralizing antibody, fresolimumab. This treatment aimed to prevent the loss of endothelial cells by inhibiting the transition from endothelial to mesenchymal states, a process stimulated primarily by TGF-β1 and, to a lesser extent, TGF-β3. The findings highlighted that while fresolimumab effectively suppresses this transition, its efficacy varies across different TGF-β isoforms, which could influence the overall success of preventing graft rejection [192]. 

## 3. Conclusions

This review described the role of TGF-β in the wound healing processes of the cornea, highlighting both its therapeutic potential and the challenges associated with its modulation. We described the complexity of the TGF-β signaling pathways and their involvement in cellular responses that are crucial for post-injury corneal recovery and the fine balance required to avoid adverse fibrotic outcomes. We detailed the dualistic nature of TGF-β’s actions—facilitating the necessary wound repair and tissue regeneration, while also posing a risk of pathological fibrosis which may lead to impaired vision due to overactivity. Novel therapeutic interventions targeting various components of the TGF-β signaling cascade have shown promise in modulating these effects. The development of monoclonal antibodies, fusion proteins, and small molecule inhibitors specifically designed to temper the TGF-β pathway offer a pathway to mitigate the fibrotic responses while enhancing healing processes. However, the clinical application of these therapies requires a nuanced understanding of TGF-β’s roles in corneal pathology to maximize benefits and minimize potential adverse effects. Future research should focus on refining these therapeutic strategies to improve specificity and efficacy, potentially through personalized medicine approaches that consider individual variations in TGF-β signaling. Additionally, exploring the interactions between TGF-β and other signaling molecules could unveil new therapeutic targets and lead to the development of combination therapies that better control wound healing processes. Ultimately, advancing our understanding of TGF-β’s function and regulation in ocular tissues will be critical in devising effective treatments for ocular surface disorders that are aimed at preserving and restoring vision.

## Figures and Tables

**Table 1:** TGF-β-based therapies developed for human use to date.

Disease	Therapy Type	Description	Status	TGF-β Receptor Focus	References
Various Cancers	Monoclonal Antibodies	Fresolimumab (GC1008) is a monoclonal antibody targeting TGF-β1-3, used in cancers like melanoma and renal cell carcinoma.	Clinical trials	TGF-βRI, TGF-βRII, TGF-βRIII	[110,111,112]
	Monoclonal Antibody	NIS793 is an anti-TGF-β monoclonal antibody, tested in combination with PD-1 inhibitors for solid tumors.	Clinical trials	TGF-β1, TGF-β2	[113,114]
Fibrosis	Small molecule inhibitors	Galunisertib (LY2157299) targets TGF-βRI to inhibit fibrosis-related pathways.	Clinical trials	TGF-βRI	[115,116,117,118,119,120]
Scleroderma	Antisense oligonucleotides	Targets TGF-β mRNA to decrease its production, potentially reducing skin thickening and organ damage.	Early research	TGF-β mRNA	[121,122,123,124]
Marfan Syndrome	Losartan	Impacts TGF-β signaling indirectly, beneficial for treating Marfan syndrome.	In use/approved	Indirect	[125,126,127,128]
Diabetic Nephropathy	Pirfenidone	Targets TGF-β-related pathways to reduce fibrosis in the kidneys.	Clinical trials	TGF-β related pathways	[129,130]
Heart Disease	ARBs	Angiotensin II receptor blockers indirectly affect TGF-β signaling involved in myocardial remodeling.	In use/approved	Indirect	[131,132,133]
Other Cancers	Kinase Inhibitors	LY2157299 and other kinase inhibitors block TGF-βRI to prevent downstream signaling, used in cancers like glioblastoma.	Clinical trials	TGF-βRI	[116,119,134]
